# Selective Detection of *Escherichia coli* K12 and *Staphylococcus aureus* in Mixed Bacterial Communities Using a Single-Walled Carbon Nanotube (SWCNT)-Functionalized Electrochemical Immunosensor with Dielectrophoretic Concentration

**DOI:** 10.3390/nano13060985

**Published:** 2023-03-08

**Authors:** Inae Lee, Heejin So, Jungyoon Kim, Joong-Hyuck Auh, Marisa M. Wall, Yong Li, Kacie Ho, Soojin Jun

**Affiliations:** 1 Department of Human Nutrition, Food, and Animal Sciences, University of Hawaii, Honolulu, HI 96822, USA; inaelee@hawaii.edu (I.L.);; 2 Department of Food Science & Technology, Chung-Ang University, Anseong 17546, Republic of Korea; 3 Daniel K. Inouye U.S. Pacific Basin Agricultural Research Center, 64 Nowelo Street, Hilo, HI 96720, USA

**Keywords:** biosensor, foodborne pathogens, microwire, impedance, rapid detection

## Abstract

An electrochemical immunosensor has been developed for the rapid detection and identification of potentially harmful bacteria in food and environmental samples. This study aimed to fabricate a microwire-based electrochemical immunosensor (MEI sensor) for selective detection of *Escherichia coli* and *Staphylococcus aureus* in microbial cocktail samples using dielectrophoresis (DEP)-based cell concentration. A gold-coated tungsten microwire was functionalized by coating polyethylenimine, single-walled carbon nanotube (SWCNT) suspension, streptavidin, biotinylated antibodies, and then bovine serum albumin (BSA) solutions. Double-layered SWCNTs and 5% BSA solution were found to be optimized for enhanced signal enhancement and nonspecific binding barrier. The selective capture of *E. coli* K12 or *S. aureus* cells was achieved when the electric field in the bacterial sample solution was generated at a frequency of 3 MHz and 20 V_pp_. A linear trend of the change in the electron transfer resistance was observed as *E. coli* concentrations increased from 5.32 × 10^2^ to 1.30 × 10^8^ CFU/mL (R^2^ = 0.976). The *S. aureus* MEI sensor fabricated with the anti-*S. aureus* antibodies also showed an increase in resistance with concentrations of *S. aureus* (8.90 × 10^2^–3.45 × 10^7^ CFU/mL) with a correlation of R^2^ = 0.983. *Salmonella typhimurium* and *Listeria monocytogenes* were used to evaluate the specificity of the MEI sensors. The functionalization process developed for the MEI sensor is expected to contribute to the sensitive and selective detection of other harmful microorganisms in food and environmental industries.

## 1. Introduction

Microbial detection assays for complex samples, such as food, water, or soil, remain a challenging task involving sample purification, discrimination of target analytes, and low-level bacteria detection. Food and environmental samples have an assortment of various components, such as organic and inorganic particles, biochemical compounds, and background microflora, that can interfere with accurate sensing assessments [1,2]. A culture-based method has been advantageous for the identification of pathogens in samples. Selective and differential media can provide both qualitative and quantitative information of microorganisms tested [3]. The culture-based method is relatively sensitive, with limited detection of 10–100 CFU/mL, and has high-level specificity. However, it takes about 24 to 72 h to obtain the results due to extra enrichment and incubation steps [4]. On the other hand, culture-independent methods such as polymerase chain reaction (PCR) have high sensitivity (limit of detection (LOD): <100 CFU/mL), despite a short assay time of approximately 1–3 h. However, these require complex instruments in stationary laboratories that can be operated by skilled personnel only [5]. The limitations of conventional detection methods point to the need for innovative technological development to rapidly detect foodborne pathogens in food products.

A novel bioaffinity and electrochemical impedance-based biosensor has been developed for rapid and simple detection and identification of target microorganisms in samples with high-level sensitivity and specificity. It can measure the change in electrical properties of electrode structures as cells become entrapped or immobilized on or near the electrode [6]. Electrochemical impedance spectroscopy (EIS) is a widely used technique for probing bioaffinity binding or biocatalytic reaction at the surface of electrodes [7] and shows the electrical responses of an electrochemical cell to sinusoidal voltage signals as a function of frequency. It enables a direct measurement to occur during the biochemical reaction on a transducer surface without secondary antibodies, enzymes, or fluorescence labels for the optical identification of analytes. Although electrochemical detection has several advantages, such as low cost, the ability to work with turbid samples, and easy miniaturization, sensitivity and selectivity are slightly limited [8]. However, immobilization of high-recognition elements such as antibodies, bacteriophages, enzymes, single-stranded DNA, or RNA can enhance the selectivity and specificity of electrochemical biosensors [9]. EIS coupled with immunology-based techniques has been used for rapid detection and qualification of foodborne pathogens [10,11]. Lu et al. [10] reported that an anti-*E. coli* antibody-immobilized microwire sensor was able to detect and enumerate *E. coli* K12 cells in suspension down to 10^3^ CFU/mL. The EIS technique was proven to be an alternative to fluorescence microscopy. Another research group fabricated a nanoporous membrane-based impedimetric immunosensor for label-free detection of pathogenic *E. coli* O157:H7 in whole milk [11]. Their system had a detection limit of 83.7 CFU/mL with 95% probability, which means that there is a 95% likelihood that *E. coli* O157:H7 at this concentration level would be detectable.

The key challenge of bioaffinity and electrochemical impedance-based biosensor fabrication is to ensure sensitive and stable bioreceptors on the sensing platform, although the antibody–antigen reactions generate sensible shifts in the electrical signal. The signal can be improved by the incorporation of nanomaterials into the biosensor [12]. Among various nanomaterials, carbon nanotubes (CNTs) have become promising materials for advanced electrochemical biosensors due to their unique properties [13,14]. CNTs offer the advantages of a large surface-to-volume ratio [12,15] and a fast electron transfer rate [13,16,17]. Their high surface area-to-weight ratio allows for more biorecognition materials to be loaded on the CNT structure. When targeted biomolecules bind to recognition materials immobilized on the CNTs, electric signals are significantly changed. Jain et al. [12] found that current density was amplified by the immobilization of SWCNTs on electrode surfaces. Yamada et al. [17] reported that a network of SWCNTs on a bio–nano combinational junction sensor enhanced the signal response sevenfold. SWCNTs modulated with polyethylenimine and biomolecules could enhance signals upon binding with *E. coli* cells.

The electrochemical immunosensor combined with dielectrophoresis (DEP) can be one of the strategies to reduce detection time and enhance sensitivity [18,19]. DEP uses the effect of the electrical polarization of particles under the influence of nonuniform electric fields to induce a translational motion [20]. The particle can be polarized under an inhomogeneous AC electric field and show two behaviors: either moving toward, called positive DEP, or repelling from, called negative DEP, the maximum electric field. The direction of polarized particle movement depends on the properties of the particle, the strength and frequency of the applied field, and the conductivity of the supporting medium [21]. DEP has been studied to electrically control trapping [22,23], manipulation [18,24], and separation [25,26] of charged particles.

In this study, it was hypothesized that a microwire-based electrochemical immunosensor (MEI sensor) assisted with positive DEP could rapidly and selectively detect specific bacteria in the presence of nonspecific bacteria. In addition, SWCNTs layered on the surface of the MEI sensor can amplify electrical detection signals when target bacteria bind to corresponding antibodies immobilized on it. There have been no reports on development of an MEI sensor conjugated with nanomaterials coupled with DEP concentrations to detect microbial cells lower than 10^3^ CFU/mL, distinguish different species or serotypes of bacteria, and separate bacteria from complex sample matrices with assay completion within 1 h. Therefore, the effects of the functionalization process on the sensor’s sensitivity and specificity for the detection of *E. coli* K12 as well as the selective detection of *E. coli* K12 and *S. aureus* in mixed bacterial communities were investigated.

## 2. Materials and Methods

### 2.1. Materials and Instruments

Seven percent (7%) gold-plated tungsten wire with a diameter of 50 μm was manufactured from ESPI metals (Ashland, OR, USA). Polydimethylsiloxane (PDMS; Sylgard 184 silicone elastomer curing agent and base) was ordered through Dow Corning (Midland, MI, USA). SWCNTs (SWNT PD1.5L) were manufactured by NanoLab Inc. (Waltham, MA, USA). Polyethylenimine (PEI, branched, average MW ~25,000), N,N-dimethylformamide (DMF), streptavidin from *Streptomyces avidinii* and bovine serum albumin (BSA; A3294) were purchased from Sigma Aldrich (St. Louis, MO, USA). Biotinylated polyclonal antibodies specific to *E. coli* (from rabbit, PA1-73031) and *S. aureus* (from rabbit, PA1-73174), and Oxoid MacConkey agar were supplied by Thermo Fisher Scientific (Waltham, MA, USA). BD Bacto^TM^ peptone, BD BBL^TM^ tryptic soy broth (TSB), BD Difco^TM^ plate count agar, 95% alcohol, and phosphate-buffered saline (PBS) were purchased from VWR (West Chester, PA, USA). Petrifilm^TM^ Staph express count plates based on Baird-Parker medium and Petrifilm^TM^ environmental listeria plates were obtained from 3M Food Safety (St. Paul, MN, USA). Platinum wire with a diameter of 0.5 mm and Ag/AgCl reference electrode for constructing the electrochemical cell was supplied by CH Instruments, Inc. (Austin, TX, USA) and VWR (West Chester, PA, USA), respectively.

SWCNTs were dispersed in DMF using a digital sonifier (450, Branson, Danbury, CT, USA). PEI, lyophilized streptavidin, and BSA were dissolved in distilled water. Both *E. coli* and *S. aureus* antibodies were tenfold diluted in PBS. Electrolyte solution used for EIS measurement was prepared by dissolving 5 mM K_3_Fe(CN)_6_ and 5 mM K_4_Fe(CN)_6_ in 0.1 M KCl solution (244023, P3289, and P9541, Sigma-Aldrich Co., Saint Louis, MO, USA).

An automated XYZ stage and stepping motor (Franklin Mechanical & Control Inc., Gilroy, CA, USA) controlled by the COSMOS program (Velmex, Inc., Bloomfield, NY, USA) was used to manipulate the microwire position during the coating step of SWCNTs and antibodies and bacterial detection. The DEP field was generated using a function generator (3220A, Agilent Technologies, Santa Clare, CA, USA). Electrochemical impedance was measured using a frequency response analyzer (μAutolab III/FRA2 potentiostat/galvanostat, Metrohm Autolab USA Inc., Riverview, FL, USA) equipped with NOVA software version 1.6.

### 2.2. Bacterial Sample Preparation

Frozen stock cultures of *E. coli* K12, *S. aureus*, *Salmonella typhimurium* (ATCC 14028), and *Listeria monocytogenes* (F2365) were provided by the Food Microbiology Lab, University of Hawaii. Each 100 μL of bacterium stock was inoculated in 10 mL of TSB twice and incubated at 35 °C for 24 h. For pure bacterial solutions, cultured bacteria were serially diluted in the 0.1% peptone water. Mixed microbial communities were prepared by transferring 100 μL of target bacteria solution (10^3^ CFU/mL) into 900 μL of nontarget bacterial dilutions with a concentration of approximately 10^4^ CFU/mL. The concentrations of the stock cultures were obtained using the plate counting method before and after the experiments. MacConkey agar, 3M Petrifilm Staph express count plates, xylose lysine deoxycholate agar, and 3M Petrifilm Listeria plates were used to enumerate *E. coil* K12, *S. aureus*, *S. typhimurium*, and *L. monocytogenes* in the cocktail sample, respectively. The concentrations of each bacterium in pure and mixed samples were summarized in Table 1. All experiments were conducted in a certificated biosafety level II laboratory.

### 2.3. Functionalization of Microwire Surface

**Figure 1:**
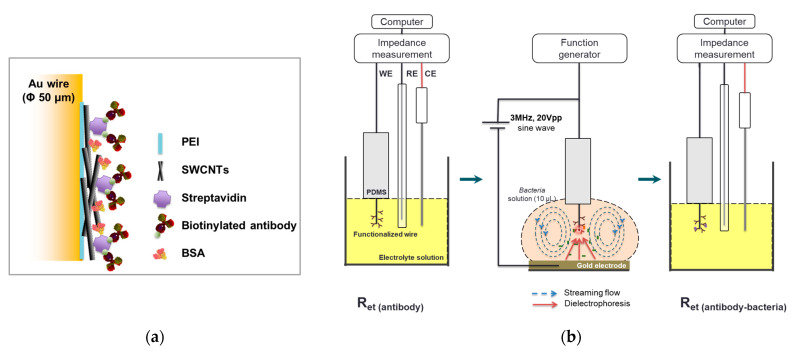
Scheme of the functionalized microwire surface (**a**) and electrochemical immunosensing with dielectrophoretic concentration (**b**).

The microwires were cut into 25 mm in length and washed with distilled water and 70% alcohol using the digital sonifier for 5 min each. The sanitized wire was functionalized with multiple layers of PEI, SWCNTs, streptavidin, antibodies, and BSA (Figure 1a). The first layer on the entire surface of the microwire was coated with 1% PEI and baked in a furnace at 175 °C for 1 h, followed by SWCNT incorporation in PEI networks using 0.01% SWCNT dispersion. The end of PEI-SWCNT-coated microwire was immersed in 5 μL of streptavidin droplet on the PDMS supporting layer for 5 min, then being withdrawn. In the same way, the droplets of antibodies and BSA were used to coat the microwire sequentially. The microwire was conjugated with specific antibodies for *E. coli* or *S. aureus* and denoted as *E. coli* MEI sensor or *S. aureus* MEI sensor. These dipping and retracting processes were repeated twice per coating step. Functionalized microwires were stored in a refrigerator before use for bacterial detection.

### 2.4. DEP-Based Concentration

A droplet of the bacterial sample (10 μL) was placed in a hemispheric concave (3 mm in diameter) on a gold plate as a bottom electrode. The functionalized microwire was dipped in the droplet at a velocity of 50 mm/min until the distance between the microwire tip and bottom electrode was as close as 1 mm. DEP was applied at 3 MHz and 20 V_pp_ [27] for 2 min; thereafter, the microwire was withdrawn at a speed of 5 mm/min for impedance measurement.

Pure *E. coli* K12 and *S. aureus* stock dilutions from 10^2^ to 10^8^ CFU/mL were used to test the sensor’s sensitivity. The specificity and selectivity of the MEI sensor for the detection of *E. coli* K12 (EC) were evaluated against pure nontarget bacteria, such as *S. aureus* (SA), *S. typhimurium* (ST), and *L. monocytogenes* (LM), and mixtures of two different bacteria (EC + SA, EC + ST, or EC + LM). In the same manner, pure EC, ST, and LM solutions and their cocktail samples mixed with *S. aureus* were used to test the specificity and selectivity of the *S. aureus* MEI sensor.

### 2.5. Impedance Measurement

**Figure 2:**
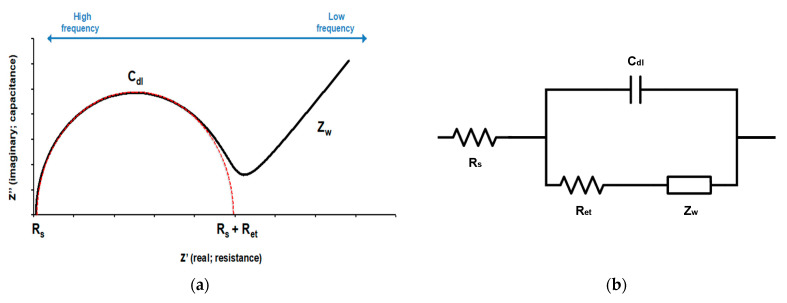
(**a**) A Nyquist plot for the electrochemical system under mixed kinetic and diffusion control and (**b**) Randles equivalent circuit used to fit impedance spectroscopy. Rs: electrolyte solution resistance, Cdl: double-layer capacitance, Ret: electron transfer resistance, and Zw: Warburg impedance.

Electrochemical impedance measurements were carried out within a frequency range of 0.1–100 kHz at a set potential of 200 mV and AC amplitude of 10 mV. The electrochemical cell consisted of a three-electrode configuration, where the MEI sensor was utilized as the working electrode, the platinum wire was applied as the counter electrode, and an Ag/AgCl electrode in 3 M KCl solution was used as the reference electrode (Figure 1b). Experimental data were displayed as Nyquist plots (Figure 2a). The Nyquist plots were fitted by the built-in analytical tool in the NOVA software. Then, electron transfer resistance (R_et_) for the redox reaction at the electrode–film interface was obtained from the equivalent circuit model (Figure 2b). The changes in electron transfer resistance (ΔR_et_) by target binding events were calculated as follows:

ΔR_et_ = R_et_ (antibody-bacteria) − R_et_ (antibody) (1)

### 2.6. Data Analysis

Microbial samples containing each serially diluted concentration of *E. coli* K12 and *S. aureus*, nontarget bacteria, and the cocktail samples were tested in triplicate. The mean and standard deviation of ΔR_et_ were calculated for each sample. The differences between the means were analyzed based on Duncan’s multiple range tests using a single-factor analysis of variance (ANOVA) offered by Statistical Analysis Software (SAS version 9.4, SAS Institute Inc., Cary, NC, USA) at a 95% confidence level (*p* ≤ 0.05).

### 2.7. FESEM Visualization and Validation

Field-emission scanning electron microscopy (FESEM, Pacific Biosciences Research Center, University of Hawaii, Model: Hitachi S-4800) was used to visualize and validate the surface of the functionalized microwire and *E. coli* K12 and *S. aureus* cells captured on the functionalized microwires by DEP. Each microwire obtained after the functionalization process and capture of the bacteria was put into 1 mL microtubes, submerged in glutaraldehyde–cacodylate fixative for 1 h, and washed in 0.1 M cacodylate buffer twice. Osmium tetroxide 1% in cacodylate buffer was used for post-fixation for 30 min. The buffer in each container was replaced by graded ethanol series (30%, 50%, 70%, 85%, 95%, and 100%) to dehydrate bacterial cells. The treated microwires were attached to carbon tapes on aluminum stubs and coated with a thin gold–palladium layer using a Hummer 6.2 sputter coater for 45 s.

## 3. Results and Discussion

### 3.1. Effect of SWCNT Coating on Signal Enhancement

**Figure 3:**
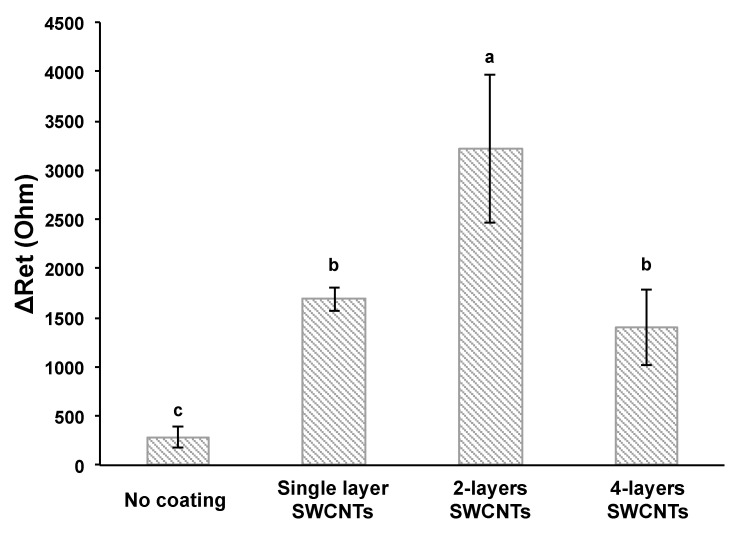
Electrical signal response enhancements by SWCNT coatings. Error bars indicate the standard deviation of the mean. Different letters (**a**–**c**) in the figure indicate significant differences (*p* ≤ 0.05).

Figure 3 shows the change in electron transfer resistance (ΔR_et_) by the antibody–bacteria reaction was increased by coating the SWCNTs on the microwire. ΔR_et_ obtained by subtracting the resistance of the antibody (R_et (antibody)_) from the resistance of the antibody–antigen (R_et (antibody-bacteria)_), indicating the resistance signals from binding *E. coli* to the *E. coli* MEI sensor. The number of SWCNT layers was controlled by the number of dip coats. Multilayered SWCNTs were fabricated by repeating SWCNT coating and drying steps. Double-layered SWCNTs on the *E. coli* MEI sensor provided signal enhancement compared to the *E. coli* MEI sensor without SWCNTs. For instance, the ΔR_et_ was 288 ± 107 Ω when the *E. coli* MEI sensor with no SWCNT layer was applied to detect the *E. coli* K12 at a concentration of 10^7^ CFU/mL. On the other hand, the values of ΔR_et_ for single- and double-layered SWCNT *E. coli* MEI sensors were 1687 ± 118 Ω and 3213 ± 748 Ω for the equivalent concentrations of *E. coli* K12, respectively. The magnitudes of ΔR_et_ were increased sixfold for the single-layered SWCNT MEI sensor and elevenfold for the double-layered SWCNT MEI sensor compared to the MEI sensor without SWCNTs. However, the resistance change decreased after the second coat.

An increase in the electric signal response can be explained by a large active electrode surface area by SWCNT coating serving as an active binding site of antibodies, permitting more antibody–antigen complexes on the electrode surface [17,28]. The modified SWCNT electrodes showed a significant increase in current density and magnitude of changes in current by binding analytes to antibodies [12,13,17]. However, more than double-layered SWCNTs would cause SWCNTs to aggregate, and the excess of SWCNTs was released from the electrode surface. This phenomenon might cause the formation of an uneven layer, i.e., the place where SWCNTs are oversaturated can affect the slowing of the electron transfer rate on the electrode surface [29].

SEM images of the bare wire right after sanitization show cracks and valley forms with high irregularity (Figure S1a,b). However, the functionalized microwire had a smooth surface with a well-developed SWCNT network, as shown in Figure S1c. When the bare wire was tested as a sensor, bacterium cells were stacked up along the cracks by capillary attraction. Undesired materials, which may interfere with electrical signal responses, were loaded on the sensor during the DEP concentration and impedance measurement. The first dip coat of PEI seemed to improve the surface structure by filling the gaps as well as modifying the surface charge for further SWCNT-coating steps. Therefore, it appears to be essential to initiate the first even and smooth coating layer to minimize the false-positive results associated with impurities and to promote appropriate layer assembly.

### 3.2. Effect of BSA Solution on Blocking Nonspecific Binding

**Figure 4:**
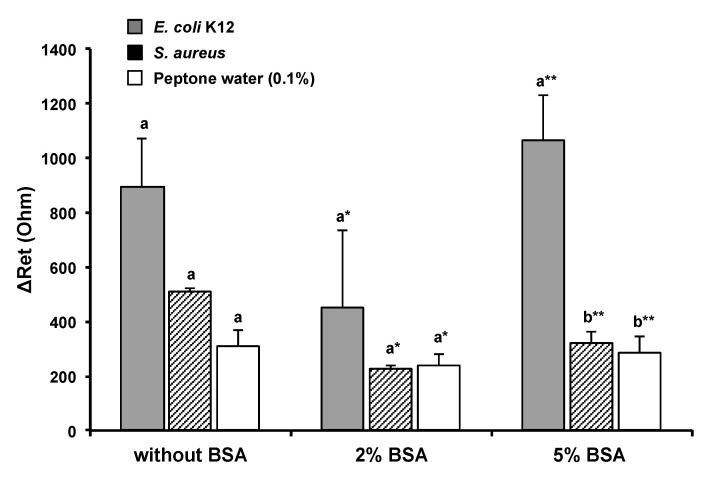
Effect of BSA on nonspecific binding reduction * and ** ΔR_et_ values from different BSA treatments were analyzed separately. Error bars indicate the standard deviation of the mean. Different letters (**a**,**b**) in the figure indicate significant differences (*p* ≤ 0.05).

Bacterial cells can be bound to immobilized antibodies via the bioaffinity reaction, but they may also be attached to the nonfunctionalized area. The latter can target bacteria or nontarget bacteria. In either case, it affects the sensor’s accuracy and sensitivity. These nonspecific binding can be minimized by filling the unoccupied sites with a blocking agent. BSA is widely used as a nonspecific binding blocker and can allow the stabilization of biomolecules bound to the surface [30,31]. In Figure 4, the response signal from cells attached to the nonfunctionalized sensing area was reduced to 58.4% by the use of 2% BSA solution. However, the *E. coli* MEI sensor coated with 2% BSA did not provide significant signal differences to discriminate *E. coli* from nontarget bacteria (*S. aureus*) and bacterium-free samples (0.1% peptone water). When the microwire was treated with 5% BSA solution, there were significant differences in ΔR_et_ between targets, nontarget bacteria, and bacterium-free samples, shown in Figure 4. The amplified electron transfer resistance change with 5% BSA treatment of the microwire might have resulted from enhanced bacteria attachments reacting with stably anchored antibody molecules.

### 3.3. Detection of E. coli K12 in Pure and Mixed Solutions

**Figure 5:**
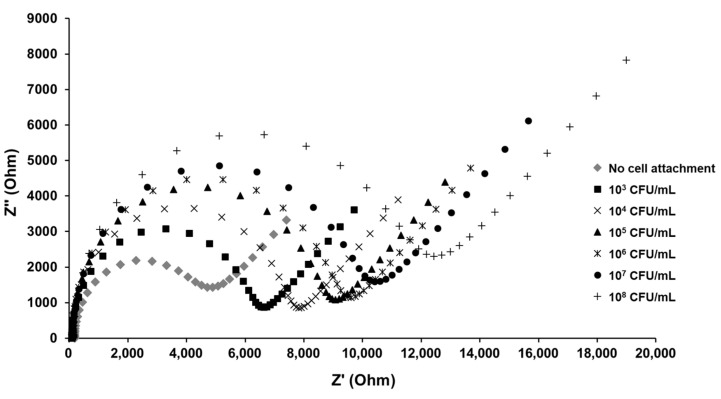
Electrochemical impedance spectra of the *E. coli* MEI sensor without *E. coli* K12 cell attachment and with *E. coli* K12 cells binding to the surfaces.

**Figure 6:**
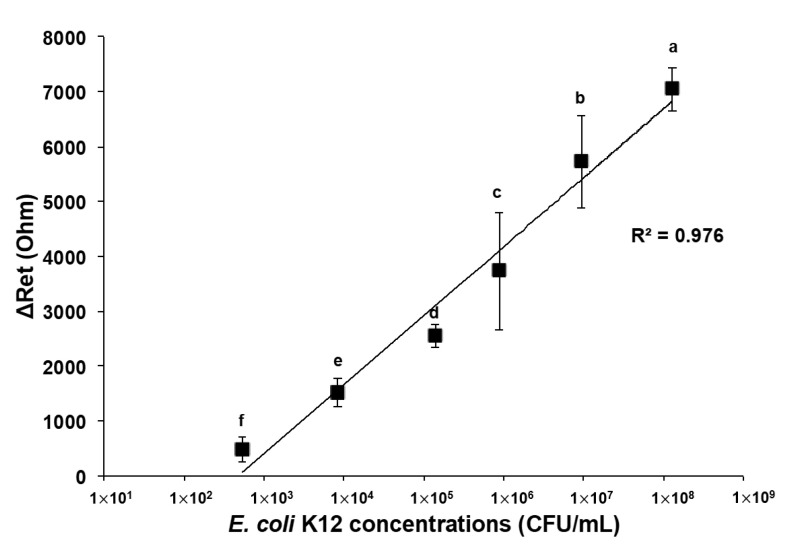
Changes in electron transfer resistance with *E. coli* K12 captured on the electrode surface of the *E. coli* MEI sensor in pure *E. coli* K12 solution. Error bars indicate the standard deviation of the mean. Different letters (**a**–**f**) in the figure indicate significant differences (*p* ≤ 0.05).

Impedance is proportionally responsive to the *E. coli* K12 concentration interacting with the surface of the *E. coli* MEI sensor, as shown in Figure 5. The diameter of the semicircle indicates that the electron transfer resistance gradually increased as the *E. coli* K12 concentration increased. The linear regression for the detection of pure *E. coli* K12 samples was observed in the range from 5.32 × 10^2^ to 1.30 × 10^8^ CFU/mL (Figure 6). Due to a small volume of each sample (10 μL) for detection, the ΔR_et_ value for the bacterial solution at cell concentrations lower than 10^2^ CFU/mL was not significant. The LOD for the *E. coli* MEI sensor was 8.21 × 10^2^ CFU/mL with a detection time of 10 min, including both cell concentration and signal measurement.

**Figure 7:**
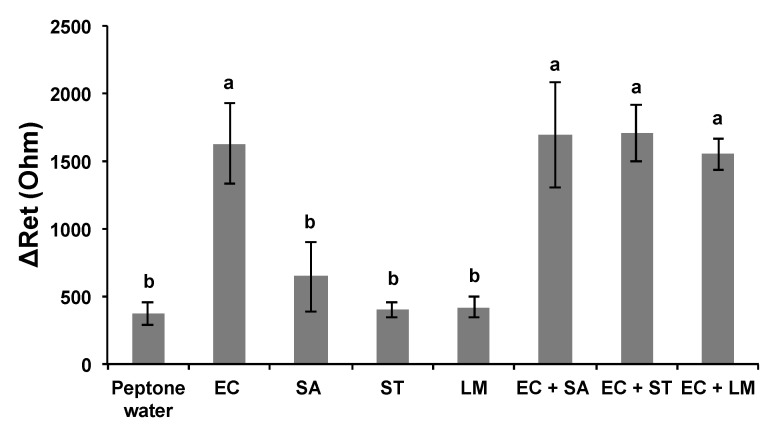
Specificity and selectivity of the *E. coli* MEI sensor for detection of *E coli* K12 against nontarget bacteria suspension and cocktail samples. Mean bacteria suspensions in pure and mixed samples. EC: *E. coli* K12, SA: *S. aureus*, ST: *S. typhimurium*, LM: *L. monocytogenes*, EC + SA: a mixture of *E. coli* K12 and *S. aureus*, EC + ST: a mixture of *E. coli* K12 and *S. typhimurium*, and EC + LM: a mixture of *E. coli* K12 and *L. monocytogenes*. Error bars indicate the standard deviation of the mean. Different letters (**a**,**b**) in the figure indicate significant differences (*p* ≤ 0.05).

**Figure 8:**
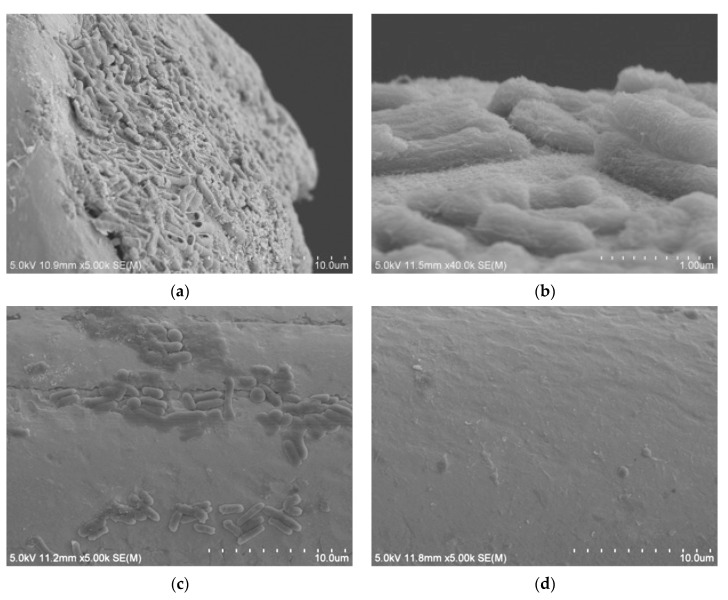
*E. coli* K12 attachment on the base plane (×5.0 k) (**a**) and the cylinder side (×40.0 k) (**b**) of microwire observed by SEM. (**c**) Bacterial attachment on the surface of the *E. coli* MEI sensor (**a**) when applied to the cocktail solution (*E. coli* K12 and *S. aureus*) and (**d**) when applied to the pure *S. aureus* solution.

When the *E. coli* MEI sensor was used for the detection of nontarget bacteria, such as SA, ST, and LM, the value of ΔR_et_ ranged between 400 and 645 Ω; however, the signal increased to 1629 ± 295 Ω when the same sensor was used for EC (Figure 7). There were no significant differences in ΔR_et_ values between pure and cocktail samples for the equivalent amount of *E. coli* K12 cells either. The values obtained for mixed samples EC + SA, EC + ST, and EC + LM were 1696 ± 390 Ω, 1702 ± 207 Ω, and 1553 ± 117 Ω, respectively, very close to the signal magnitude for EC sample. SEM images present captured *E. coli* K12 on the surface of the *E. coli* MEI sensor (Figure 8a,b). Most of the bacteria attached to the surface of the *E. coli* MEI sensor were observed as *E. coli* K12 cells after the *E. coli* MEI sensor was tested with the mixture of *E. coli* K12 and *S. aureus*, shown in Figure 8b. FESEM also indicated that the surface of the *E. coli* MEI sensor remained clear, which means no bacteria were captured under DEP when the sensor was dipped into the pure *S. aureus* solution (Figure 8c).

These electrical signal response results agreed with Jain et al. [12] reporting that decreased current density and increased impedance were observed due to the formation of antibody–antigen (*Salmonella*) complexes on the glassy carbon electrode immobilized with SWCNTs. Antibody–bacteria complexes attached to the surface and insulating properties of the cell walls of the bacteria slow the electron transfer rate within electrochemical cells, resulting in increased electron transfer resistance [12,32].

The role of DEP for bacteria immunosensing is to form a nonuniform electric field in solution, leading the bacterial cells to the location of the antibodies. It has been reported that the configuration of the microwire electrode and the flat electrode has a high surface-to-area ratio difference and forms a strong electric field near the microwire [23]. In an environment conditioned to be directed towards a strong electric field (applied voltage, frequency, charge in solution, etc.), bacteria move near microwires and bind to antibodies on the surfaces. Positive DEP-driven bacterial cell trapping for detection has been reported for *Salmonella* and *E. coli*. *S. typhimurium* was concentrated on the detector of the sensor chip at 100 kHz and 10 V_pp_ for 40 min [24]. They achieved the rapid and sensitive detection of *S. typhimurium* in deionized water and artificially contaminated mineral water samples with LODs of 56 CFU/mL and 110 CFU/mL, respectively. *E. coli* K12 was deposited directly on the electrode array in a droplet (1 μL) form by applying 1 MHz and 5 V_pp_ into the electrode device [33]. Other researchers found that *E. coli* K12 displayed positive DEP behavior and was captured by the antibody-immobilized microwire at 20 V_pp_ and 1–10 MHz. At 3 MHz, the fluorescence intensity and impedance were significantly increased due to increases in the antibody and bacterial antigen complexes [10,23]. The electrochemical immunosensor with dielectrophoretic attraction for bacteria detection has the advantage of concentrating microorganisms present in a low number of bacterial cells in the sample to the detection part to improve detection sensitivity and to process small volumes in a short time

### 3.4. Developed Electrochemical Immunosensor for Detection of S. aureus in Pure and Mixed Solutions

**Figure 9:**
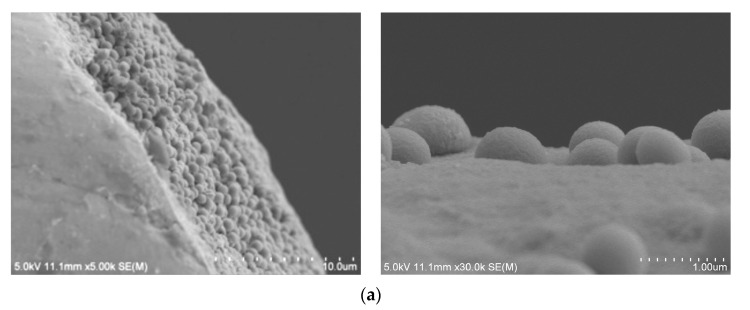

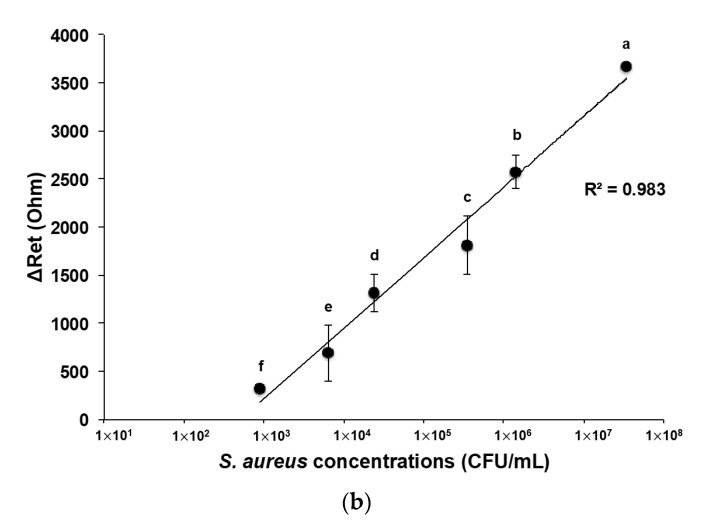
(**a**) SEM images of *S. aureus* bacteria cells on the base plane (left, ×5.0k) and the cylinder side (right, ×30.0k) of the microwire. (**b**) Changes in electron transfer resistance with *S. aureus* captured on the electrode surface of the *S. aureus* MEI sensor in pure solution. Error bars indicate the standard deviation of the mean. Different letters (a–f) in the figure indicate significant differences (*p* ≤ 0.05).

**Figure 10:**
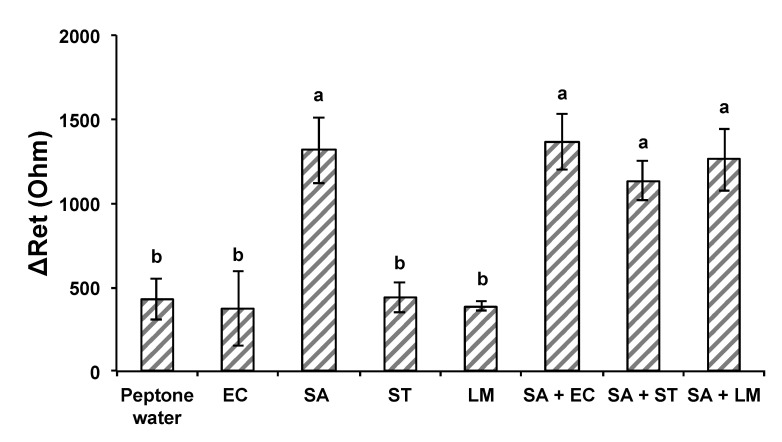
Specificity and selectivity of the *S. aureus* MEI sensor against nontarget bacteria and cocktail samples. Bacteria suspensions in pure and mixed samples. EC: *E. coli* K12, SA: *S. aureus*, ST: *S. typhimurium*, LM: *L. monocytogenes*, SA + EC: a mixture of *S. aureus* and *E. coli* K12, SA + ST: a mixture of *S. aureus* and *S. typhimurium*, and SA + LM: a mixture of *S. aureus* and *L. monocytogenes*. Error bars indicate the standard deviation of the mean. Different letters (**a**,**b**) in the figure indicate significant differences (*p* ≤ 0.05).

An anti-*S. aureus* antibody layer was introduced to fabricate another MEI sensor for the detection of *S. aureus*. The attachment of *S. aureus* cells on the *S. aureus* MEI sensor’s surface could be observed in the SEM images as well (Figure 9a). The electrical signal response of *S. aureus* MEI sensor shows a similar pattern that was obtained from the *E. coli* MEI sensor, as indicated in Figure 9b. The ΔR_et_ was increased as *S. aureus* concentration increased from 8.90 × 10^2^ to 3.45 × 10^7^ CFU/mL with a R^2^ value of 0.983. The LOD for the *S. aureus* MEI sensor was determined to be 8.90 × 10^2^ CFU/mL. The specificity of the *S. aureus* MEI sensor was also validated using nontarget bacteria, EC, ST, and LM (Figure 10). The ΔR_et_ values of nontarget bacteria were 373 ± 220 Ω, 440 ± 88 Ω, and 389 ± 30 Ω. However, the magnitudes of ΔR_et_ estimated from microbial cocktail samples mixed with *S. aureus* (SA + EC, SA + ST, and SA + LM) were not statistically different from signal SA detection.

These findings demonstrated that MEI sensors for bacterial detection can be fabricated by simply changing the antibodies specific to target analytes. It should be noted that the functionalization procedure and bionanomaterial layers used in this study enabled the stable bioreceptor platforms on the MEI sensor to selectively detect the bacteria.

## 4. Conclusions

The goal of this study was to develop a bio- and nanomaterial-functionalized electrochemical immunosensor-assisted DEP and fluidic technology for rapid and reliable detection of potentially harmful microorganisms in food. The MEI sensor functionalized with double-layered SWCNTs and 5% BSA solution provided sensitive and specific detection of target bacteria. Functionalization contributed to signal enhancement and cell-binding specificity, as changes in electrical signal were proportional to bacterial concentrations. The rapid (within 10 min) and sensitive detection of *E. coli* K12 and *S. aureus* in pure bacterial samples were achieved at the LOD of 10^3^ CFU/mL. The developed MEI sensor shows potential for selective detection of target bacteria in mixed bacterial communities. It can be used for microbial analysis of complex samples in food and environmental industries. Future studies should be conducted to extend our understanding of the pathogen detection mechanism with electrochemical immunosensor and to accomplish better detection efficiency. The DEP effect on the bacterial concentration in buffer solution was also captured adequately in this study; however, more knowledge about bacterial cell behaviors is required for the DEP manipulation of bacterial cells in more highly conductive food samples than buffers. The optimization of the sensing parameters of medium conductivity, applied voltage and frequency, and DEP experience time for efficient separation of bacteria and viruses from food samples can be explored in further studies. Efficient DEP conditions, such as device-specific electrode configuration and electroactive sample loading size based on the comprehensive understanding of complex effects on the microbial particles in the DEP field, should be investigated.

## Figures and Tables

**Table 1:** Concentrations of each bacterium in pure and in mixed samples when *E. coli* MEI sensor (top) and *S. aureus* MEI sensor (bottom) were tested for specific and selective detection.

Concentration	In Pure Sample (CFU/mL)	
Bacteria	Target	Nontarget
*E. coli* K12	8.53 × 10^3^	
*S. aureus*		1.41 × 10^4^
*S. typhimurium*		1.60 × 10^4^
*L. monocytogenes*		3.40 × 10^4^
Concentration	In Mixed Sample (CFU/mL)	
Bacteria	Target	Nontarget
*E. coli* K12 *+ S. aureus*	1.67 × 10^4^	9.76 × 10^4^
*E. coli* K12 *+ S. typhimurium*	1.15 × 10^4^	3.30 × 10^3^
*E. coli* K12 *+ L. monocytogenes*	1.90 × 10^4^	9.00 × 10^2^
Concentration	In Pure Sample (CFU/mL)	
Bacteria	Target	Non-Target
*E. coli* K12		1.96 × 10^4^
*S. aureus*	2.39 × 10^4^	
*S. typhimurium*		3.70 × 10^4^
*L. monocytogenes*		1.59 × 10^4^
Concentration	In Mixed Sample (CFU/mL)	
Bacteria	Target	Nontarget
*S. aureus + E. coli* K12	5.65 × 10^4^	2.33 × 10^4^
*S. aureus + S. typhimurium*	6.08 × 10^3^	9.08 × 10^3^
*S. aureus + L. monocytogenes*	9.83 × 10^3^	2.20 × 10^4^

## Data Availability

Not applicable.

## Supplementary Materials

The following supporting information can be downloaded at https://www.mdpi.com/article/10.3390/nano13060985/s1. Figure S1: SEM images for bare (a,b) and functionalized (c,d) microwires.
