# Supplementary figures and images for: Selective Detection of Escherichia coli K12 and Staphylococcus aureus in Mixed Bacterial Communities Using a Single-Walled Carbon Nanotube (SWCNT)-Functionalized Electrochemical Immunosensor with Dielectrophoretic Concentration
Source: Nanomaterials (Basel). 2023 Mar 8;13(6):985. doi: 10.3390/nano13060985 (PMC10051117; doi:10.3390/nano13060985)

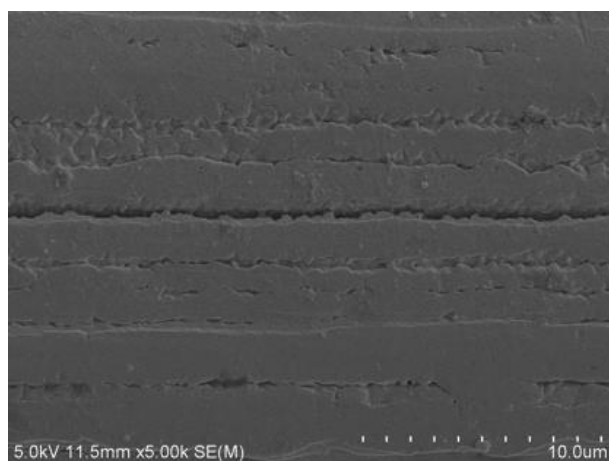

(a)

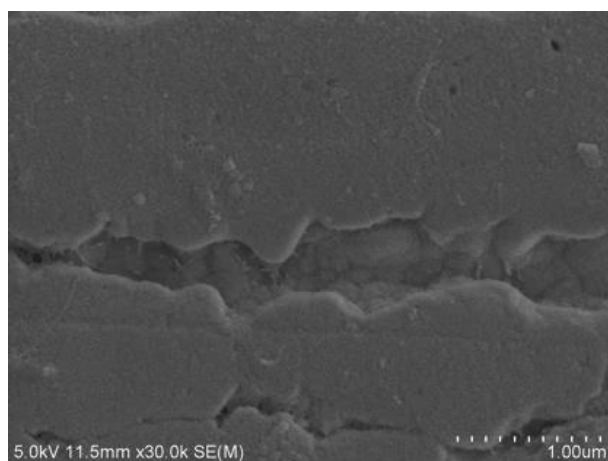

(b)

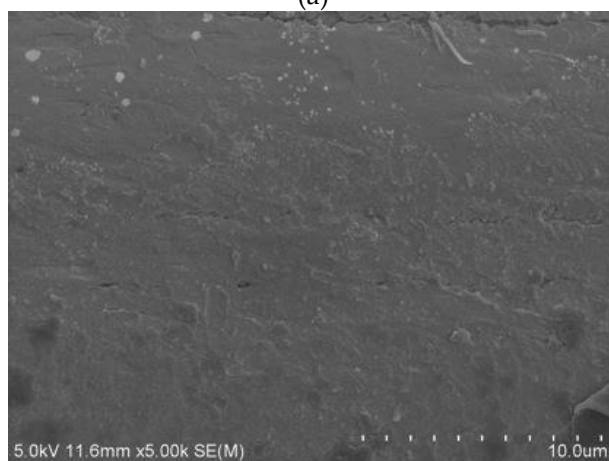

(c)

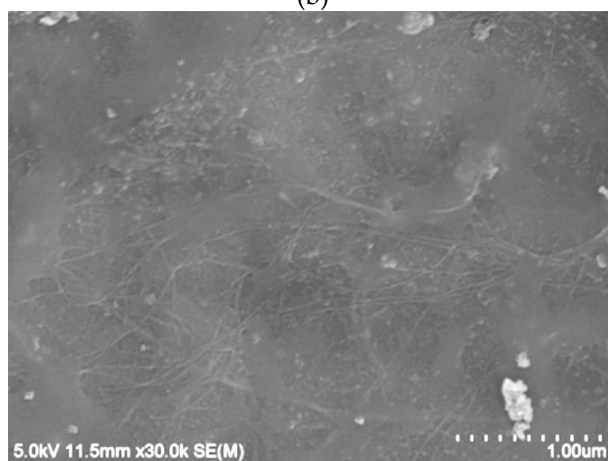

(d)

**Figure S1.** SEM images for bare (a)-(b) and functionalized (c)-(d) microwires

Supplement: Supplementary file 1 [file nanomaterials-13-00985-s001.zip › nanomaterials-2238874-supplementary.pdf]
